# Metabolic Profiling of Alpinetin in Rat Plasma, Urine, Bile and Feces after Intragastric Administration

**DOI:** 10.3390/molecules24193458

**Published:** 2019-09-24

**Authors:** Jieying Qiu, Hongyu Wu, Feng Feng, Xiaoying He, Caihong Wang, Shenghui Chu, Zheng Xiang

**Affiliations:** School of Pharmaceutical Sciences, Wenzhou Medical University, Wenzhou 325035, China; qiujieying0109@163.com (J.Q.); pop_coorn@163.com (H.W.); FF000999@163.com (F.F.); hxy17858505013@163.com (X.H.); wangch2017@126.com (C.W.)

**Keywords:** alpinetin, UPLC-Q-TOF-MS, metabolic profiling, rats

## Abstract

Alpinetin, a bioactive flavonoid, has been known to have a diverse therapeutic effect, with namely anti-inflammatory, anticancer and antioxidant effects with low systemic toxicity. This study aimed to obtain metabolic profiles of alpinetin in orally administrated rats. The metabolites of alpinetin were systematically analyzed and identified by ultra-performance liquid chromatography quadrupole time-of-flight mass spectrometry (UPLC-Q-TOF-MS). The chromatographic separation was achieved on a High Strength Silica (HSS) T3 (1.8 μm, 2.1 × 100 mm) column with the mobile phase consisting of water containing 0.1% formic acid and acetonitrile with 0.1% formic acid via gradient elution. An extracted ion chromatogram strategy based on multiple prototype/metabolite intermediate templates and 71 typical metabolic reactions was proposed to comprehensively profile the metabolites of alpinetin. With the metabolite profiling strategy, altogether 15 compounds were recognized from urine, plasma, bile and feces of rats after intragastric administration of alpinetin for the first time. The prototype, glucuronide conjugates and phenolic acids metabolites were the probable predominant form of alpinetin in rats. This work showed a comprehensive study of the probable metabolic pathways of alpinetin in vivo, which could provide meaningful information for future pharmacological studies.

## 1. Introduction

*Alpinia katsumadai* Hayata seeds, known as CaoDouKou in China, have been used in traditional Chinese medicine for thousands of years to cure digestive and inflammatory diseases [1]. Alpinetin is known as a natural flavonoid, and was firstly extracted from *Amomum subulatum* Roxb’s seed. It is considered as the primary active ingredient of Zingiberaceae (*Alpinia katsumadai* Hayata) [2]. Modern pharmacological research shows a series of pharmacological activities that are involved in alpinetin, including anti-inflammatory [3,4,5,6], anti-cancer [7,8,9,10], anti-oxidant effects [11]. Alpinetin was found to improve the sensitivity of drug-resistant lung cancer cells to cis-diammined dichloridoplatium [10], which may be used as a potential drug for combination therapy. Recently, it has been reported that alpinetin may be deemed as a good candidate for some possible treatment of liver injury and brain diseases [12,13]. Because of its multiple therapeutic activities, alpinetin is considered as a potential candidate drug for further clinical studies, and has recently attracted more and more attention from medical and pharmaceutical fields [14,15,16]. Therefore, a better understanding of the efficacy of alpinetin needs to elucidate its biological fate in the body.

For an active component of natural medicine, the substance basis of pharmacological action may include not only the prototype drug component but also the metabolite formed by the biotransformation of the prototype compound [17]. Due to profound conjugative metabolism, alpinetin, which is known as a flavonoid with one hydroxyl group, tends to be featured with poorer oral bioavailability [18,19]. Previous metabolism studies found that the glucuronide conjugates are the major metabolites of alpinetin, but the biological and pharmacological activity of the glucuronide conjugated metabolites may be completely different from that of the prototype [20]. This suggests that not only alpinetin itself, but also its metabolites may be responsible for its numerous pharmacological effects. Therefore, the exploring of the metabolic characteristics of alpinetin is of great significance. To our knowledge, there are no relevant reports about the metabolic characteristics of alpinetin. In this study, the biological samples (plasma, urine, bile and feces) after intragastric administration of alpinetin were analyzed by ultra-high performance liquid chromatography quadrupole time-of-flight mass spectrometry (UPLC-Q-TOF-MS). The structures of metabolites were further characterized by high resolution mass (HRMS) and MS/MS fragment (MS^n^) data. We hope that some meaningful information for the enlightenment of future pharmacological researches of alpinetin will be provided by the results.

## 2. Experimental Section

### 2.1. Materials

Alpinetin (99.0% purity) was purchased from Chengdu Munster Biotechnology Co., Ltd. (SChuan, China). A Milli-Q system (Millipore, Bedford, MA, USA) prepared the ultra-pure water. Methanol, HPLC-grade formic acid, and acetonitrile were purchased in Sigma-Aldrich Co (St. Louis, MO, USA). Further, the other reagents and chemicals were of analytical grade.

### 2.2. Drug Administration and Animals

Male Sprague–Dawley (SD) rats (200 ± 20 g) were purchased from the Experimental Animal Center of Wenzhou Medical University (Wenzhou, China). All experimental procedures were approved by the Animal Ethics Committee in Wenzhou Medical University (No. WMU2018-0983). The animals were maintained in an environmentally controlled breeding room before experiments at a temperature of 23–25 °C and 45–55% humidity and then fasted for 12 h prior to the experiment.

Twelve rats were divided into four groups (three rats per group): Plasma, urine, bile and feces groups. The rats were given alpinetin by intragastric administration at a dose of 40 mg/kg. Bile samples were collected from bile intubated rats prior to dose and 0–4 h, 4–8 h, 8–12 h, 12–24 h, 24–36 h after administration. The samples of urine and feces were collected 4 h pre-dose and 0–12 h, 12–24 h, 24–36 h post-dose. Approximately 150 μL blood samples were collected from the heparinized tail vein at 0, 1, 5, 15, 30, 45 min, and 1, 3, 5, 8, 12, 24, 36 h after administration. Furthermore, the samples of plasma were acquired by samples of centrifuging blood for 10 min at 4500 rpm. All the plasma samples were mixed and pooled for the time points of 1–15 min, 30–60 min, 3–8 h, and 12–36 h across rats in equal volumes. Bile, urine, and feces samples were pooled at each time period in equal volumes or weights. All biological samples were frozen at −20 °C before analysis.

### 2.3. Instrument and UPLC-Q-TOF-MS Conditions

The analysis was performed combined with a system of Waters I-Class UPLC (Waters Corporation, Milford, USA) on a system of Waters Xevo G2-XS Q-TOF. The UPLC separation was used held at 40 °C by an ACQUITY UPLC HSS T3 column (1.8 μm, 2.1 × 100 mm), with the flow rate of 0.2 mL/min. The mobile phase consisted of solvent A (water containing 0.1% formic acid) and solvent B (acetonitrile with 0.1% formic acid). The gradient elution was performed as follows: 0–2 min, 95% A; 2–10 min, 95–80% A; 10–13 min, 80–70% A; 13–22 min, 70–15% A; 22–25 min, 15% A; 25–25.5 min, 15–95% A; 25.5–30 min, 95% A. The sample injection volume was 3 μL.

The MS experiments were performed by the positive mode via an electrospray ionization (ESI) interface. The following sets show the optimal MS parameters: Source temperature, 100 °C; capillary voltage, 2.5 KV; cone gas flow, 50 L/h; cone voltage, 40 V; desolvation gas flow, 800 L/h. Compounds were detected by precursor ions and fragment ions using elevated collision energies (MS^E^) centroid analysis from *m*/*z* 100–1200 Da at a resolving power of 30,000 with a scan time of 0.2 s. Nitrogen was used as cone gas, and argon as collision gas. Collision-induced dissociation was performed with low-energy and high-energy functions. In the low-energy function, collision energy was off to acquire HRMS data; in the high-energy function, a collision energy ramp 20–40 V was used to obtain MS^n^ data.

### 2.4. Sample Preparation

The protein precipitation method was performed to pre-treated samples. Pooled plasma (2 mL), urine (1 mL), and bile samples (1 mL) were extracted with methanol (10 mL) in test tubes. Freeze-dried pooled fece samples were milled and weighed (100 mg). Each fece sample was ultrasonically extracted with methanol (1 mL) for 60 min. After extraction, all biological samples were vortexed for 5 min and centrifuged at 4500 rpm for 10 min. The supernatants were evaporated in a nitrogen stream at 30 °C to dryness. The residues were dissolved in methanol of 100 μL and vortexed for 5 min. The sample was then centrifuged at 13,000 rpm for 10 min, and 10 μL of the supernatant was injected into the UPLC-Q-TOF-MS system for analysis.

### 2.5. Data Analysis

The data of UPLC-Q-TOF-MS were obtained and processed with MassLynx Version 4.1 software (Waters Co.). The process of metabolite profiling was divided into two steps. Firstly, an in-house developed software was designed to predict expected alpinetin metabolites automatically. This method used alpinetin and its metabolic intermediate as templates, inputted their chemical formula, calculated the accurate high resolution mass spectrometric data with the ionization set as [M + H]^+^ and [M − H]^−^, and predicted expected alpinetin metabolites based on 71 typical template metabolic reactions. Secondly, screening and validation of potential metabolites by extracted ion chromatogram was performed with mass tolerance set to 5 ppm, and MS^2^ confirmation manually.

## 3. Results

### 3.1. Mass Fragmentation of Alpinetin

Drug metabolism is a process of structural modification of drugs through enzymatic systems in vivo and in vitro. Since the prototype compounds and their metabolites are similar in chemical structure, it is necessary to study the mass spectrometric decomposition of prototype compounds before studying the metabolites of alpinetin.

The alpinetin was detected in plasma, urine, bile and feces, which was eluted at 16.52 min. It exhibited an accurate [M + H]^+^ ion at *m*/*z* 271.0943 (C_16_H_15_O_4_). The fragment ion at *m*/*z* 257.1538 resulted from a fragment ion *m*/*z* 271.0943 by the loss of -CH_2_. Fragmentation ions of *m*/*z* 104.0645, and 167.0298 were produced by the Retro Diels–Alder reaction, which is a typical cleavage method for flavonoids. The MS^2^ spectra showed a characterized fragmentation pathway of *m*/*z*: 167.0297→151.0581→123.1169 (with a loss of -O and -CO, respectively). Comparison of the retention time between P and the alpinetin standard further indicated that P was alpinetin.

### 3.2. Identification of Metabolites

The biological samples (plasma, urine, bile and feces) of alpinetin were analyzed by way of the UPLC-Q-TOF-MS method. The biological samples of alpinetin and blank samples were obtained and processed with MassLynx Version 4.1 software. Firstly, 71 typical metabolic reactions were incorporated in an in-house developed software. Multiple templates were selected based on an extensive literature review and experience-rich structural analysis to generate a list of potential metabolites. Following that, potential metabolites would further be validated with multiple-dimension data, including the extracted ion chromatogram (EIC) strategy (HRMS data), mass difference match (HRMS data), and fragment confirmation (MS^n^ data). The maximum mass errors between the measured and calculated values were <5 ppm. With the metabolite profiling strategy, a total of 14 metabolites were identified from biological samples after intragastric administration of alpinetin (Table 1).

M1 was detected in urine and bile. An accurate [M + H]^+^ ion at *m*/*z* 255.1017 (C_16_H_15_O_3_) showed in M1 with the retention time of 14.98 min, 15.9913 Da less than that of alpinetin. The characteristic fragment ions at *m*/*z* 151.0358 and 104.0659 derived from precursor ion *m*/*z* 255.1017 by the Retro Diels–Alder reaction, which suggested that M1 was the deoxidation metabolite of the parent. The fragment ion at *m*/*z* 136.0713 resulted from the fragment ion *m*/*z* 151.0358 by the loss of -O.

Metabolite M2 presented in plasma and urine, which was detected at 13.41 min with a protonated molecule [M + H]^+^ ion at *m*/*z* 273.1131 (C_16_H_17_O_4_), 2.0183 Da more than that of alpinetin. The M2 was identified as a reduction product of alpinetin. The characteristic fragments at *m*/*z* 104.0751 and 169.0380 derived from the precursor ion *m*/*z* 273.1131 by the Retro Diels–Alder reaction. The fragment ion at *m*/*z* 241.0980 resulted from the fragment ion *m*/*z* 273.1131 by the loss of -OCH_3_. Characteristic fragments at *m*/*z* 225.1078 were formed by the loss of -H_2_O from precursor ion *m*/*z* 243.0980.

In bile, urine and feces, M3 was found. Metabolite M3 exhibited an accurate [M + H]^+^ ion at *m*/*z* 287.0929 (C_16_H_15_O_5_) with the retention time of 13.28 min, 15.9981 Da less than that of alpinetin. Characteristic fragments at *m*/*z* 271.0908 were formed by the loss of -O from precursor ion *m*/*z* 287.0929. The fragment ion at *m*/*z* 255.1097 resulted from fragment ion *m*/*z* 287.0929 by the loss of -OCH_3_. The fragment ion at *m*/*z* 167.0414 and 104.0494 resulted from *m*/*z* 271.0908 by the Retro Diels–Alder reaction. Therefore, M3 was elucidated as the oxidized metabolite of parent.

M4 was detected in urine, feces and bile, which was eluted at 15.29 min with a protonated molecule [M + H]^+^ ion at *m*/*z* 285.0735 (C_16_H_13_O_5_), 2.0194 Da less than that of M3. This revealed that M4 was the desaturated product of M3. The fragment ions at *m*/*z* 269.1329 were due to *m*/*z* 285.0727 by the loss of -O. The fragment ion at *m*/*z* 253.1393 resulted from the fragment ion *m*/*z* 285.0727 by the loss of -OCH_3_. The characteristic fragment ions at *m*/*z* 118.0619 and 167.0116 derived from precursor ion *m*/*z* 285.0727 by the Retro Diels–Alder reaction.

M5 was found in urine and feces. Metabolite M5 showed a protonated molecule [M + H]^+^ ion at *m*/*z* 313.1051 (C_18_H_17_O_5_) with the retention time of 15.47 min, which was 42.0103 Da higher than that of alpinetin. Fragment ions at *m*/*z* 271.0935 were generated by the loss of -CH_3_CO from the precursor ion *m*/*z* 313.1051. The fragment ions at *m*/*z* 104.0014 and 167.0921 resulted from *m*/*z* 271.0935 by the Retro Diels–Alder reaction. The characteristic fragment ions at *m*/*z* 209.1138 derived from precursor ion *m*/*z* 313.1051 by the Retro Diels–Alder reaction. Therefore, M5 was considered the acetylated metabolite of alpinetin.

M6 was detected in urine and bile, which was detected at 15.69 min with a protonated molecule [M + H]^+^ ion at *m*/*z* 328.1169 (C_18_H_18_O_5_N), 57.0221 Da more than that of alpinetin. Fragment ions at *m*/*z* 271.0935 were produced by the loss of -NH_2_CH_2_CO from fragment ion *m*/*z* 328.1169. Fragment ions at *m*/*z* 257.0778 were generated by the loss of -CH_3_ from precursor ion *m*/*z* 271.0935. The fragment ions at *m*/*z* 104.0637 and 167.0298 derived from the precursor ion *m*/*z* 271.0935 by the Retro Diels–Alder reaction. Therefore, M6 was elucidated as the glycine conjugates of parent.

Metabolite M7 was only found in urine. Metabolite M7 showed a [M + H]^+^ ion at *m*/*z* 367.0467 (C_16_H_15_O_8_S), which was 79.9538 Da more than that of M3. Therefore, M7 was a sulfation product of M3. The fragment ions at *m*/*z* 353.2278 derived from precursor ion *m*/*z* 367.0467 by the loss of -CH_3_. Fragment ions at *m*/*z* 287.0929 were generated by the loss of -SO_3_ from precursor ion *m*/*z* 367.0467. The characteristic fragment ions at *m*/*z* 271.0935 came from fragment ion *m*/*z* 367.0464 by the loss of -SO_3_ and –O, respectively. The characteristic fragment ions at 104.0638 and 167.0297 derived from precursor ion *m*/*z* 271.0935 by the Retro Diels–Alder reaction. The fragment ions at *m*/*z* 167.0297 and 120.0632 were created from *m*/*z* 287.0929 by the Retro Diels–Alder reaction.

M8 was found in plasma, urine and bile, which was eluted at 13.94 min with a [M + H]^+^ ion at *m*/*z* 447.1283 (C_22_H_23_O_10_), 176.0335 Da more than that of alpinetin. This revealed that M8 was the glucuronide conjugated product of alpinetin. The characteristic fragment ions *m*/*z* 271.0944 were generated by the loss of -C_6_H_8_O_6_ from precursor ion *m*/*z* 447.1283. The fragment ions at *m*/*z* 104.0646 and 167.0298 derived from *m*/*z* 271.0944 by the Retro Diels–Alder reaction.

M9 was only found in urine, which was detected at 15.39 min with a protonated molecule [M + H]^+^ ion at *m*/*z* 463.1230 (C_18_H_17_O_5_), 176.030 Da more than that of M3. Characteristic fragment ions at *m*/*z* 287.0878 were due to fragment ion *m*/*z* 463.1230 by the loss of -C_6_H_8_O_6_. The characteristic fragment ions at *m*/*z* 271.0940 were produced by the loss of -O from precursor ion *m*/*z* 287.0878. The fragment ions at *m*/*z* 104.0714 and 167.0226 were produced from *m*/*z* 271.0940 by the Retro Diels–Alder reaction. M9 was elucidated as the glucuronide conjugated metabolite of M3.

M10 only presented in feces, which was eluted at 11.79 min. Metabolite M10 showed a [M + H]^+^ ion at *m*/*z* 257.0806 (C_15_H_13_O_4_), which was 14.0142 Da less than that of alpinetin. Therefore, M10 was the demethylated metabolite of alpinetin. The typical ions *m*/*z* 241.1519 were produced from a precursor ion *m*/*z* 257.0869 by the loss of -O. The fragment ions at *m*/*z* 104.0504 and 153.0302 resulted from *m*/*z* 257.0869 by the Retro Diels–Alder reaction.

M11 was only found in feces. M11 eluted at 15.96 min, which exhibited a [M + H]^+^ ion at *m*/*z* 273.0741 (C_15_H_13_O_5_), 14.0188 Da less than that of M3. This result indicated that M11 was a demethylated metabolite of M3. Fragment ions of *m*/*z* 257.1676 were produced from fragment ion *m*/*z* 273.0730 by the loss of -O. The typical ions *m*/*z* 241.1911 were produced from precursor ion *m*/*z* 257.1878 by the loss of -O. The fragment ions at *m*/*z* 152.0578 derived from precursor ion *m*/*z* 273.0741 by the Retro Diels–Alder reaction.

M12 only presented in feces, which was detected at 11.21 min with a protonated molecule [M + H]^+^ finding suggested that M12 was the hydration metabolite of alpinetin. The fragment ions *m*/*z* 271.1481 emerged by the loss of -H_2_O from the precursor ion *m*/*z* 289.1072. Fragment ions of *m*/*z* 255.1161 were produced from fragment ion *m*/*z* 271.1481 by the loss of -O. The fragment ions at *m*/*z* 104.0451 and 167.1030 derived from the precursor ion *m*/*z* 271.1481 by the Retro Diels–Alder reaction.

M13 was found in urine and feces, which was eluted at 15.55 min with a [M + H]^+^ ion at *m*/*z* 351.0520 (C_16_H_15_O_7_S), 79.9572 Da less than that of alpinetin. This result indicated that M13 was sulfate conjugated metabolite of alpinetin. Fragment ions of *m*/*z* 333.2450 produced from fragment ion *m*/*z* 351.0520 by the loss of -H_2_O. The ions *m*/*z* 255.2070 were produced from the precursor ion *m*/*z* 333.2450 by the loss of -SO_3_.

M14 was detected in plasma, urine, bile and feces, which was eluted at 2.36 min. It exhibited an accurate [M + H]^+^ ion at *m*/*z* 166.0468 (C_9_H_10_O_3_). It has been reported that flavonoids can be transformed into low-molecular-weight phenolic acids by intestinal bacteria. The fragment ions at *m*/*z* 148.0353 derived from precursor ion *m*/*z* 166.0468 by the loss of -H_2_O. Fragment ions of *m*/*z* 104.0660 produced from fragment ion *m*/*z* 148.0353 by the loss of -COO. Therefore, M14 was elucidated as the phenolic acids converted by the intestinal bacteria.

**Figure 1:**
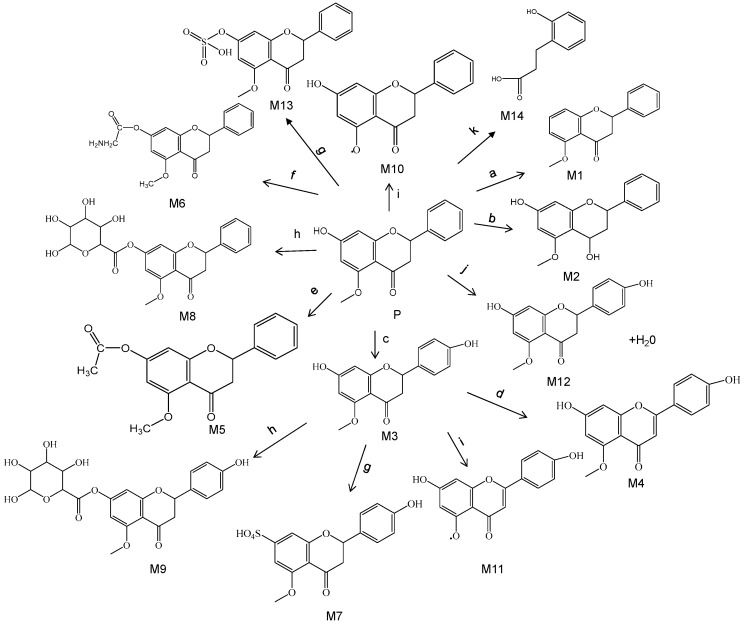
Proposed metabolic pathway of alpinetin in rats after oral administration. (**a**. Deoxydation; **b**. Reduction; **c**. Oxidation; **d**. Desaturation; **e**. Acelylation; **f**. Glycine conjugation; **g**. Sulfation; **h**. Glucuronide conjugation; **i**. Demethylation; **j**. Hydration; **k**. cleavage).

**Figure 2:**
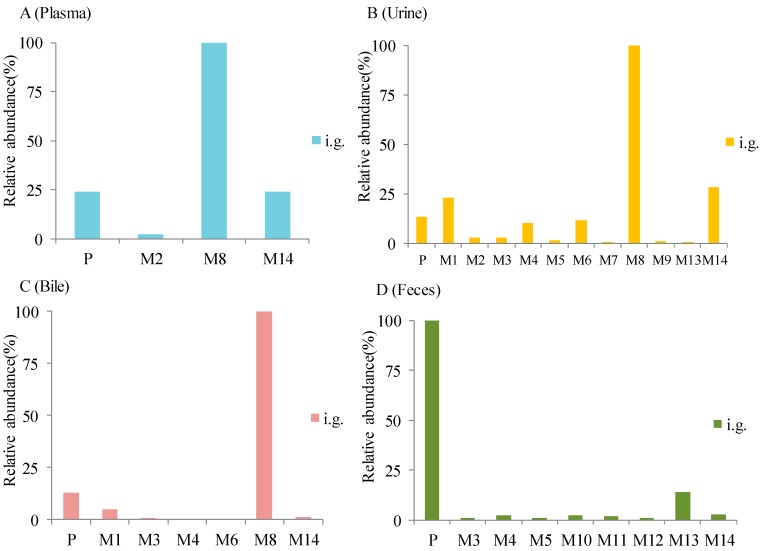
Metabolite profile in plasma (**A**), urine (**B**), bile (**C**), and feces (**D**) after administration of alpinetin. Relative abundances are expressed by the percentage of MS response with the most abundant compound being 100.

**Figure 3:**
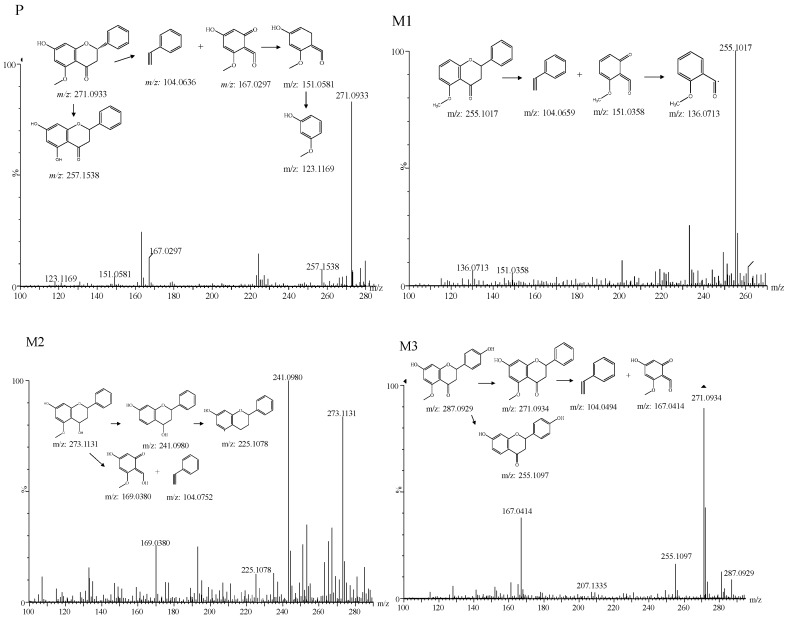

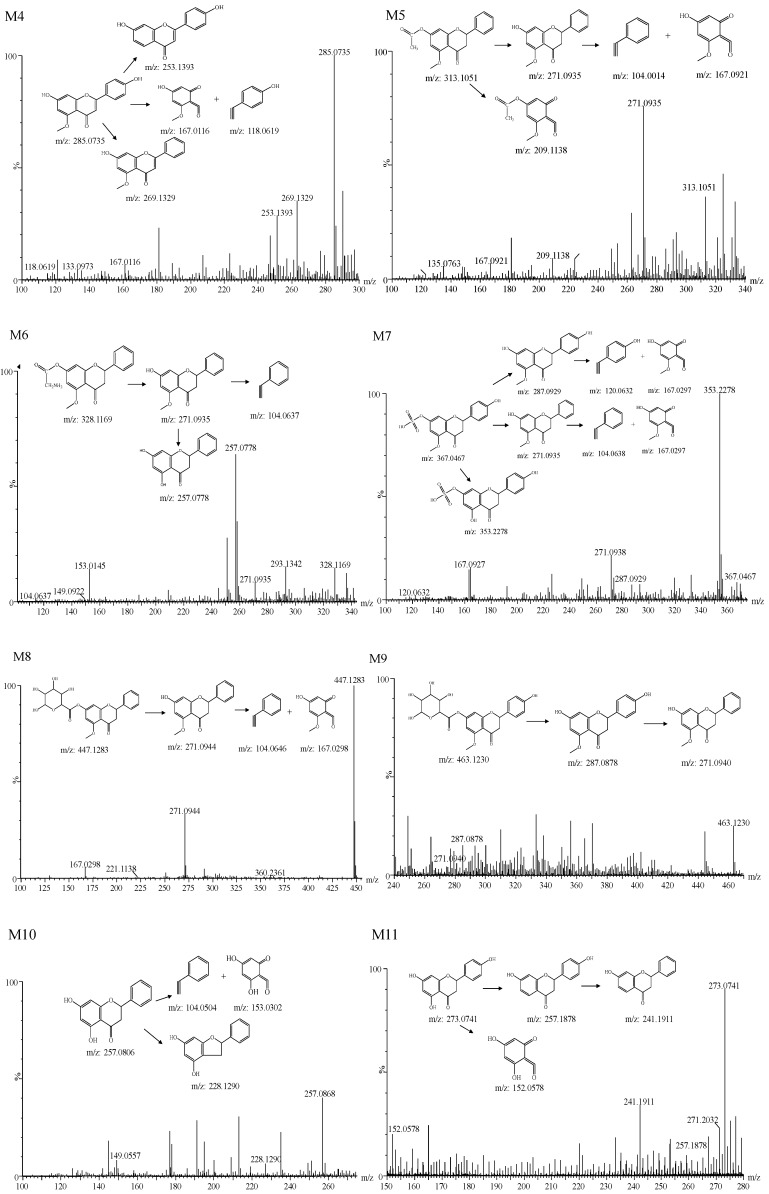

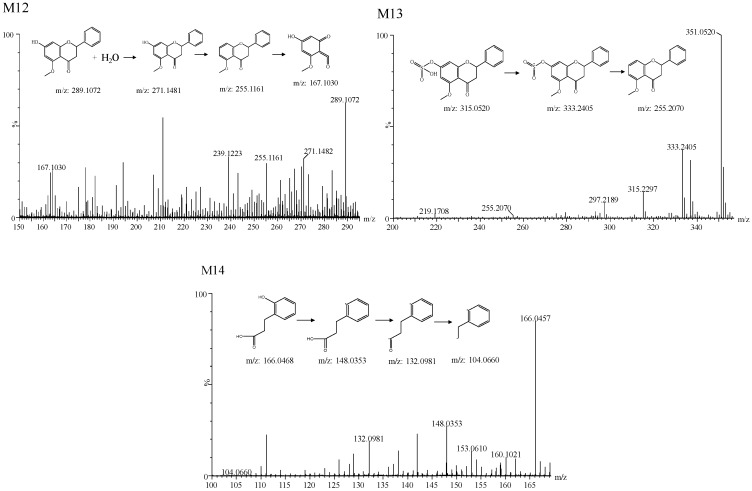
Structure and the MS^2^ spectrum of the alpinein and its probable metabolites.

The proposed metabolic pathways are summarized in Figure 1. For 14 metabolites, M2, M8, M14 were detected in plasma, M1–M9, M13, M14 were detected in urine, M1, M3, M4, M6, M8, M14 were presented in bile, while M3, M4, M5, M10, M11, M12, M13, M14 were detected in feces. As shown in Figure 2, the prototype, glucuronide conjugates and phenolic acids metabolites were the predominant form of alpinetin in rats. The structure and the MS^2^ spectrum of the alpinetin and its probable metabolites are shown in Figure 3. This work showed a comprehensive study of the probable metabolic pathways of alpinetin in vivo. Future work will focus on the synthesis of newly identified major metabolites and assess their biological activity and toxicity.

## 4. Discussion

Alpinetin, a bioactive flavonoid, has been known to have diverse therapeutic effects, namely anti-inflammatory, anticancer and antioxidant effects. The main objective of our study was to analyze the metabolites of alpinetin in rats. Alpinetin, which is known as a flavonoid with one hydroxyl group, tends to be characterized with poorer bioavailability due to a profound conjugative metabolism [21]. We demonstrated in this study that significant glucuronidation was undergone by alpinetin in rats. It was noteworthy that the glucuronide conjugates could be detected in rat plasma, urine and bile. This was supported by the fact that intestine-specific enzyme uridine diphospho-glucuronosyltransferase1A10 (UGT1A10) is characterized with the poorest ability to catalyze alpinetin glucuronidation [18]. It has been reported that the UGT1A1, 1A3, 1A9 and 2B15 were determined to participate in the alpinetin glucuronidation in human liver microsomes [20]. Therefore, due to the prevalence of genetic polymorphism in UGT enzymes, individuals with different polymorphisms may exhibit different metabolic activities of alpinetin.

In our study, the metabolites of alpinetin can be explained by eleven proposed pathways: Deoxidation, reduction, oxidation, desaturation, acetylation, glycine conjugation, sulfation, glucuronidation, demethylation, hydration and cleavage. In recent years, more and more attention has been paid to the metabolic process of flavonoids [22]. The metabolic pathways of flavonoids mainly include conjugation, cleavage and oxidation in vivo [23,24], which is consistent with the conclusions of the metabolic characteristics of alpinetin in our study. However, to date, there are no systematic and detailed data on alpinetin metabolites. This study enriched the known metabolic types of alpinetin and made the study of alpinetin metabolism more comprehensive.

From our current studies, major metabolites in the biosamples were different from each other. It was found that the prototype, glucuronide conjugates and phenolic acids were the predominant compounds of alpinetin in rat plasma. The predominant metabolites of alpinetin in rat urine and bile were the prototype, glucuronide conjugates, deoxidated and phenolic acids metabolites. The prototype and sulfate conjugates were the main metabolites of alpinetin in rat feces. It can be preliminarily speculated that alpinetin was absorbed into the blood in the small intestine of rats as prototype and phenolic acids. The absorbed prototype was glucuronidated in the blood and liver to form glucuronide conjugates and converted into bile or blood circulation. Finally, the prototype, deoxidated metabolites, glucuronide conjugates and sulfate conjugates were excreted through feces and urine.

## 5. Conclusions

This study aimed to obtain metabolic profiles of alpinetin in plasma, urine, bile and feces of the rats after intragastric administration. With the metabolite profiling strategy, altogether 15 metabolites were detected, including 12 metabolites in urine, 7 metabolites in bile, 9 metabolites in feces and 4 metabolites in plasma. The prototype, glucuronide conjugates and phenolic acids metabolites were the predominant form of alpinetin in rats. This research provides meaningful information for future pharmacological studies on alpinetin, and leads to a better understanding of the bio-transformations and the pharmaceutical applications of alpinetin.

## Figures and Tables

**Table 1:** Mass spectral data of alpinetin (P) and its metabolites (M1–M12).

NO.	RT	*m*/*z*	Formula	Polarity	Error (×10^−6^)	MS^2^	Metabolism
P	16.52	271.0948	C_16_H_15_O_4_	POS	−3.27	257,167,104	Prototype
M1	14.98	255.1017	C_16_H_15_O_3_	POS	0.39	151,136,104	Deoxydation
M2	13.41	273.1131	C_16_H_17_O_4_	POS	3.30	241,225,169,104	Reduction
M3	13.28	287.0929	C_16_H_15_O_5_	POS	5.23	271,255,104,167	Oxidation
M4	15.29	285.0735	C_16_H_13_0_5_	POS	−4.07	269,253,167,118	Oxidation + desaturation
M5	15.47	313.1051	C_18_H_17_0_5_	POS	−0.64	271,209,104,167	Acelylation
M6	15.69	328.1169	C_18_H_18_0_5_N	POS	−3.35	271,257,104,167	Glycine conjugation
M7	16.65	367.0467	C_16_H_15_O_8_S	POS	−4.08	353,287,271,104,120,167	Oxidation + sulfation
M8	13.94	447.1283	C_22_H_23_O_10_	POS	−0.67	271,104,167	Glucuronide conjugation
M9	15.39	463.1230	C_22_H_23_O_11_	POS	−1.08	287,271,104,167	Oxidation + glucuronide conjugation
M10	11.79	257.0806	C_15_H_13_O_4_	POS	−0.78	228,104,153	Demethylation
M11	15.96	273.0741	C_15_H_13_O_5_	POS	−4.22	255,241,152	Oxidation + demethylation
M12	11.21	289.1072	C_16_H_17_O_5_	POS	0.34	271,255,104,167	Hydration
M13	15.55	351.0520	C_16_H_15_O_7_S	POS	1.34	333,255	Sulfation
M14	2.36	166.0468	C_9_H_10_O_3_	POS	−2.56	148,132,104	Phenolic acids

POS, positive mode; MS^2^, MS/MS fragment.
